# 

**Published:** 1811-09

**Authors:** 


### MONTHLY CATALOGUE OF MEDICAL BOOKS.

Quincy's Lexicon Medicum. A new Medical Dictionary, containing an Explanation of the terms in Anatomy, Physiology, Practice of Physic, Materia Medica, Chemistry, Pharmacy, Surgery, Midwifery, and the Various Branches of Natural Philosophy connected with Medicine. Selected, arranged, and compiled from the best authors. By Robert Hooper, M. D. Longman aud Co.

Popular Directions for the Treatment of the Diseases of Women and Children. By John Burns, 8vo. Longman and Co.

The London Dispensatory, containing the Elements and Practice of Materia Medica and Pharmacy, with a Translation of the Pharmacopœias of the London, the Edinburgh, and the Dublin Colleges of Physicians: many useful Tables; and Copper-plates of the Pharmaceutical Apparatus; the whole forming a Synopsis of Materia Medica and Therapeutics. By Anthony Todd Thomson, Surgeon. 8vo. Longman and Co.

An Essay on the Uterine Hæmorrhage which precedes the delivery of the full-grown fœtus; illustrated with cases; fifth Edition. By Edward Rigby esq. F. L. S. 8vo. Johnson and Co.

Practical Remarks on Insanity; to which is added a Commentary on the Dissection of the Brains of Maniacs, with some account of Diseases incident to the Insane. By Bryan Crowther. 8vo. Underwood.

---

In September will be published, Fasciculus 2d, of Anatomico-Chirurgical Views, intended to illustrate the Anatomy of the Cavity of the Male and Female Pelvis and their contents, the Muscles of the Perineum as they appear upon Dissection, and the external organs of Generation in both subjects; together with appropriate explanations and references. By John James Watt.

---

### NOTICES TO CORRESPONDENTS.

A Correspondent, who signs Medicus, desires to be informed of the process for dissolving the Elastic Gum (Caoutchoc) in æther.

We have been favoured by a gentleman who signs himself a "Constant Reader, but a New Correspondent" with the following recipe for Ring-worm. R. Ferr. Sulph. ʒss. Aq. Rosæ ℥j. M. ft. Lotio. This applied to the part two or three times in the day, our correspondent has found to be a never-failing remedy.

Mr. Thomas F. Rance requests through the medium of the Medical Journal, to thank Mr. Hamilton of Ipswich for his communication on the subject of Scarlatina, in which he recommends venæsection; it accords with the principles of practice which Mr. Rance has found most beneficial, and when a suitable case occurs, he purposes to adopt it.

We have received Communications from Dr. Ramsbotham, Messrs. Woodham, an Essex Practitioner, J. H. J.—Mr. Sur's Letter, acknowledged in a former Number, cannot be found.

---

*Printed by E. Hemsted, Great New Street, Fetter Lane.*

